# Congenital Tongue Base Cyst Presenting with Laryngeal Stridor in Youth: A Case Report

**DOI:** 10.1155/2012/147503

**Published:** 2012-08-16

**Authors:** Zouheir Zaki, Naouar Ouattassi, Noureddine Alami

**Affiliations:** ENT Head and Neck Surgery Department, Hassan II University Hospital, 30000 Road Sidi Hrazem, BP 1893 Fez, Morocco

## Abstract

*Introduction*. Tongue base cyst is an uncommon but potentially dangerous cause of stridor in neonates and infants. *Case Presentation*. We report a case of a 2-month-old Arabic male infant with a congenital tongue base cyst revealed by inspiratory stridor and recurrent respiratory distress. Diagnosis of cyst was suspected at endoscopy and confirmed by MRI imaging. The cyst was marsupialized with CO_2_ laser. One year later, the child remains asymptomatic without recurrence of the mass. *Conclusion*. Tongue base cysts should be considered in differential diagnosis in new borns with stridor, respiratory difficulties, or swallowing problems. Definitive therapy requires large marsupialization under general anesthesia.

## 1. Introduction

Congenital tongue base cysts are an uncommon cause of airway obstruction [1, 2]. An approximate annual incidence of 1.82 per 100000 live births in an oriental population was reported [3]. A tongue base cyst may cause stridor or respiratory distress or be totally asymptomatic. When the cyst is suspected clinically, endoscopic laryngoscopy and CT or MRI are necessary for diagnosis. The large laser marsupialization has become the standard therapy by most otolaryngologists.

## 2. Case Presentation 

**Figure 1:**
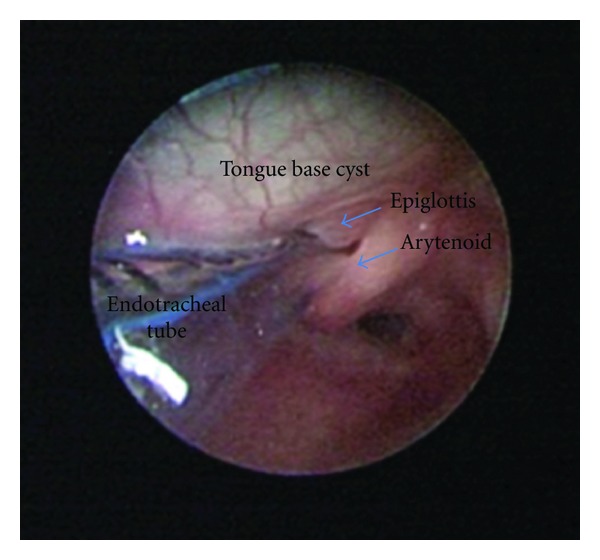
Endoscopic view showing the tongue base cyst and its contact with the larynx.

**Figure 2:**
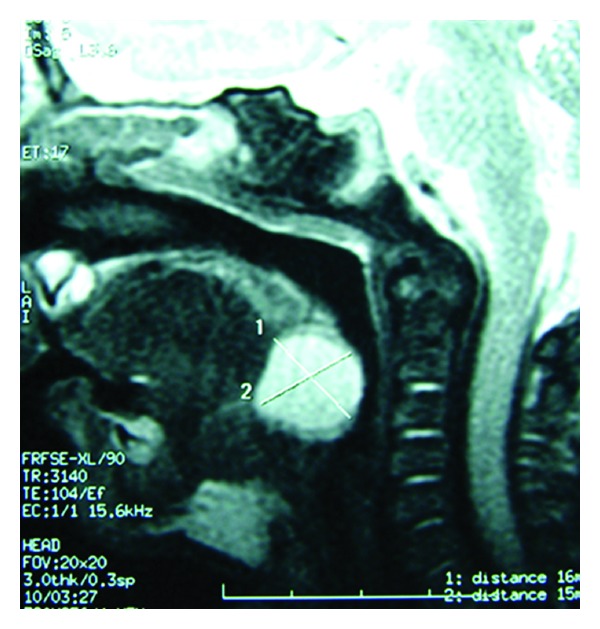
MRI in sagittal view shows a large cystic mass at the tongue base.

A 2-month-old male infant of Arabic origin and average socioeconomic statusliving inan urban area with a history of recurrent respiratory distress was referred to pediatric emergency for inspiratory stridor and sever dyspnea. Clinical examination found an apyretic child with polypnea and features of respiratory collapse, so he was intubated and transferred to pediatric intensive care. Biological analysis showed normal complete blood values, with no inflammatory syndrome, also the chest X-ray was normal. The diagnosis of laryngomalacia was suspected. The endoscopic laryngobronchoscopy was performed and disclosed a normal appearing larynx with no major inspiratory collapsus. The digital tongue base palpation revealed a submucosal soft midline mass (Figure 1). MRI showed a cystic lesion of the tongue base that drove back the epiglottis (Figure 2). Under general anesthesia, large laser CO_2_ marsupialization was done. One year after surgery, the patient was in perfect health without any airway problems and had gained weight.

## 3. Discussion

In medical literature, a variety of terms have been used for tongue base cysts, such as epiglottic cyst, lingual cyst, vallecular cyst, or laryngeal cyst [1–8]. Two major hypotheses to explain the pathogenesis are that this cyst is a consequence of either ductal obstruction of mucus glands or an embryological malformation [2]. Histologically, the cyst contains respiratory epithelium with mucous glands, with an external lining of squamous epithelium [2–4]. Most affected infants have symptoms during the first week of life [5]. Clinical manifestations consist of various degrees of upper airway obstruction such as inspiratory stridor, chest retraction, apnea, cyanosis, and feeding difficulty. Stridor is the most common symptom [1–8]. About 60% of children with stridor have laryngeal obstruction such as laryngomalacia, vocal cord paralysis, subglottic stenosis, hemangioma, or laryngeal cysts; 25% have lesions in the upper airway, including choanal atresia, macroglossia, and/or facial anomalies; 15% are due to tracheobronchial lesions such as tracheomalacia or vascular compression or other lesions [2]. In neonatal stridor, evaluation of the airway anatomy and differential diagnosis from other causes of stridor are important to prevent any mortality and morbidity from these sources. Lingual cysts are variable in size. If large, they can encroach upon the airway and displace the epiglottis, causing airway obstruction. Small cysts are totally asymptomatic. Finger palpation for a tongue base mass in children with stridor and swallowing disturbance is simple and may be the first clue to lingual cysts. However, finger palpation of the base of the tongue must be performed with great care and should be done in a controlled environment for emergency airway management. The palpation may sometimes cause rupture of the cyst [1–3]. In a young infant with respiratory stridor and dysphagia, which rapidly resolve after airway manipulations, spontaneous rupture of a tongue base cyst should be highly suspected and vigilant followup is necessary in case of recurrence [1]. Primary diagnostic approach to laryngeal or vallecular cysts should be a flexible nasopharyngeal laryngoscopy or bronchoscopy. CT and MR imaging often help narrow the differential diagnosis such as lingual thyroid, proximal cystic dilatation of the thyroglossal duct, lymphangioma or hemangioma, dermoid cyst, lipoma, fibroma, or carcinoma [6]. Although surgical removal may be the treatment of choice, other modalities such as endoscopic marsupialization, excision, and deroofing of the cyst have been recently developed. Marsupialization under general anesthesia is a safe and definitive procedure, especially when performed by CO_2_ laser. Sometimes, preintubation aspiration becomes necessary before the insertion of the endotracheal tube. Simple aspiration of the cyst is not advised because of its high recurrence rate [1–6]. Spontaneous disappearance of a tongue base cyst after oropharyngeal suctioning has not been previously reported [1–8]. In conclusion, tongue base cysts should be considered in differential diagnosis in new borns with stridor, respiratory difficulties, or swallowing problems. An endoscopic laryngobronchoscopy has to be performed before making the diagnosis of laryngomalacia. Definitive therapy requires large marsupialisation under general anesthesia. 

## References

[1] Wong KS, Huang YH, Wu CT (2007). A vanishing tongue-base cyst. *The Turkish Journal of Pediatrics*.

[2] Yang MA, Kang MJ, Hong JN (2008). A case of congenital vallecular cyst associated with GER presenting with stridor, feeding cyanosis and failure to thrive. *Korean Journal of Pediatrics*.

[3] Ahrens B, Lammert I, Schmitt M, Wahn U, Paul K, Niggemann B (2004). Life-threatening vallecular cyst in a 3 month-old infant: case report and literature review. *Clinical Pediatrics*.

[4] Hsieh WS, Yang PH, Wong KS, Li HY, Wang ECR, Yeh TF (2000). Vallecular cyst: an uncommon cause of stridor in newborn infants. *European Journal of Pediatrics*.

[5] Sands NB, Anand SM, Manoukian JJ (2009). Series of congenital vallecular cysts: a rare yet potentially fatal cause of upper airway obstruction and failure to thrive in the newborn. *Journal of Otolaryngology*.

[6] Chung PS, Chung YW, Park SJ (2004). A clinicopathologic study of epiglottic and vallecular cysts. *Korean Journal of Otorhinolaryngology*.

[7] Yao TC, Chiu CY, Wu KC, Wu LJ, Huang JL (2004). Failure to thrive caused by the coexistence of vallecular cyst, laryngomalacia and gastroesophageal reflux in an infant. *International Journal of Pediatric Otorhinolaryngology*.

[8] Gluckman PGC, Chu TWF, Van Hasselt CA (1992). Neonatal vallecular cysts and failure to thrive. *Journal of Laryngology and Otology*.

